# Impact of multidisciplinary tumour boards (MTB) on the clinicopathological characteristics and outcomes of resected colorectal liver metastases across time

**DOI:** 10.1186/s12957-020-01984-8

**Published:** 2020-09-03

**Authors:** Lionel Chen, Nicholas L. Syn, Brian K. P. Goh, Peng Chung Cheow, Prema Raj, Yexin Koh, Alexander Chung, Ser Yee Lee, London Lucien Ooi, Chung Yip Chan, Jin Yao Teo

**Affiliations:** 1 Department of Hepatopancreatobiliary and Transplant Surgery, Singapore General Hospital, Outram Rd, Singapore, 169856 Singapore; 2 Yong Loo Lin School of Medicine, National University of Singapore, Singapore, Singapore; 3 Duke-NUS Graduate Medical School, Singapore, Singapore

**Keywords:** Colorectal liver metastases, Hepatectomy, Oncology

## Abstract

**Background:**

Resection of colorectal liver metastases (CLM) has been established as the standard of care. This study aims to compare the change in clinicopathological characteristics of patients who underwent curative resection of CLM across two time periods—2000 to 2010 (P1) and 2011 to 2016 (P2) and evaluate the prognostic impact of these characteristics on survival outcomes.

**Methods:**

Patients who undergo liver resection for CLM at Singapore General Hospital from January 2000 to December 2016 were identified from a prospectively maintained database. The primary end point was overall survival.

**Results:**

There were 183/318 (57.5%) patients and 135/318 (42.5%) patients in P1 and P2, respectively. There was a lower proportion of patients who had nodal metastases from primary colorectal cancer and clinical risk score (CRS) less than 3 in P2 when compared to P1. There was no difference in survival between both time periods. Independent predictors of survival for the cohort were CEA levels ≥ 200 ng/ml, primary tumour grade and lymph nodal status. Independent predictors of poor survival in P1 were poorly differentiated colorectal cancer and nodal metastases while in P2, independent predictors of poor survival were multiple liver metastases and nodal metastases.

**Conclusion:**

Nodal metastases from primary colorectal cancer are an independent predictor of poor survival across time for resectable CLM. Although there is no difference in survival between the two time periods, patients with multiple liver metastases should be carefully considered prior to surgery as it is also an independent predictor of overall survival.

## Introduction

Colorectal cancer (CRC) imposes a heavy burden on healthcare and is a leading cause of cancer death worldwide [1]. In Asia, the incidence of CRC has increased by as much as two- to four-fold in the past few decades, and in some countries is now the most common cancer diagnosed [2]. About 25% of patients will present with stage IV CRC (synchronous metastases), and up to 50% of patients overall will go on to develop metachronous liver metastases [3, 4]. While the 10-year survival rate for patients with stage I CRC is about 90%, less than 10% of patients with non-resectable stage IV disease will survive 5 years [3].

Twenty to thirty percent of patients with stage IV CRC will have potentially resectable metastases confined to the liver. In these patients, liver resection with curative intent may be performed, with substantially improved 5-year survival rates ranging between 16 and 71% being reported [5–8].

Well-established independent predictors of disease recurrence and survival after hepatic resection include stage of the primary tumour, preoperative carcinoembryonic antigen (CEA) levels, hepatic tumour size, number of hepatic metastases, time from treatment of primary tumour to diagnosis of hepatic metastases and presence of extrahepatic disease [9]. Extensive research has been conducted on the prognostic value of Fong’s clinical risk score [5], and this has been validated in several studies. However, the treatment of colorectal liver metastases has advanced over the last two decades with developments in chemotherapy, surgical technology and also careful patient selection with greater emphasis on multi-disciplinary tumour boards.

Therefore, this paper aims to compare the change in the clinicopathological characteristics of patients who underwent curative resection of colorectal liver metastases across two time periods—2000 to 2010 (P1) and 2011 to 2016 (P2) and evaluate the prognostic impact of these clinicopathological characteristics on the survival outcomes between the two time periods.

## Methodology

Patients who had undergone liver resection with curative intent for colorectal liver metastases in Singapore General Hospital from Jan 2000 to Dec 2016 were identified from a prospectively maintained database. Patients with primary colorectal adenocarcinomas were included. Patients with extrahepatic metastases, multiple recurrences or second primary tumours were excluded. Patient demographics, primary tumour and CLM clinicopathological characteristics were collected. Pre-operative imaging included cross-sectional computer tomographic (CT) scans of the abdomen and pelvis both at the time of the diagnosis of CLM and the initial diagnosis of the colorectal primary. All patients had normal pre-operative liver function tests and coagulation profile prior to liver resection. The study was approved by the institutional review board.

The two time periods 2000 to 2010 (P1) and 2011 to 2016 (P2) were decided with the development of our institution’s dedicated multi-disciplinary tumour boards from 2011 onwards. Synchronous liver metastases were defined as the presence of liver lesions detected at the time of diagnosis of colorectal cancer. The number and size of liver lesions were determined on preoperative cross-sectional imaging, predominantly contrast-enhanced CT or magnetic resonance imaging (MRI) scans. The extent of liver resection was defined based on Brisbane 2000 nomenclature [10]. Major liver resection was defined as resection of 3 or more segments. Fong’s clinical risk score (CRS) for colorectal liver metastases was calculated. All liver resections were performed by attending surgeons from the Department of Hepatopancreatobiliary and Transplant Surgery, Singapore General Hospital, and all surgeons had adequate experience in liver resection. The standard follow-up protocol at outpatient visits every 3 to 6 monthly included clinical examination, serum carcinoembryonic antigen (CEA) level and contrast-enhanced CT scan (chest, abdomen and pelvis).

Separate survival analyses were conducted for the two study periods. Differences in patient baseline characteristics between the two time periods were compared using chi-square tests for binary variables and Student’s *t* test or Mann-Whitney *U* test for normally and non-normally distributed continuous variables, respectively. Overall survival was calculated from the time of hepatic resection until death, and patients who were alive at their last follow-up visit were censored.

Prior to analyses, missing covariate data points were multiply imputed (*M* = 50 imputations) using multivariate chained equations, with augmented logistic regression for dichotomous variables and predictive mean matching (five *k*-nearest neighbours) for continuous variables. Hazard ratios (HRs) were computed by fitting Cox proportional hazard models to each imputed dataset and subsequently combined according to Rubin’s rules.

To assess if there was a change in prognostic impact of a given covariate, interaction between the study period and the magnitude of the hazard ratio was evaluated by testing the equality of Cox model regression coefficients obtained at the two time periods using the *mi testtransform* command in Stata version 16 (StataCorp, TX, USA). For this analysis, nominal interaction *p* values of less than 0.05 (two-sided) were considered to indicate that the prognostic impact of a given covariate had changed significantly between the two time periods.

In a separate analysis, we sought to identify factors that were independently associated with overall survival within each of the two time periods. This was accomplished by fitting two Cox proportional hazards model (one for each time period) using a stepwise backward elimination procedure at a *p* threshold of < 0.10 in SPSS version 26.0 (SPSS Inc., Chicago, IL).

## Results

We identified 318 patients for this analysis (mean age, 60.6 ± 10.8 years; 187 [58.8%] men and 131 [41.2%] women). There were 183/318 (57.5%) patients and 135/318 (42.5%) patients in P1 and P2, respectively. The clinicopathological characteristics of the patients in both time periods are described in Table 1. There was no statistical difference between the 2 time periods with regard to clinical characteristics such as age, gender, American Society of Anaesthesiologists (ASA) score, gender and CEA levels. There was no difference in primary colorectal cancer pathological characteristics such as T-stage, location of the primary colorectal cancer and also the characteristics of the liver metastases such as size, distribution and number of liver metastases.

**Table 1:** Baseline patient demographics and clinicopathological characteristics (*n* = 318)

Baseline variables	2000–2010 (***n*** = 183)	2011–2016 (***n*** = 135)	***p*** value
Age at resection, years			
Median (IQR)	60.2 (52.2–67.8)	62.6 (55.7–68.3)	0.1354
Sex			
% males	105/183 (57.4%)	82/135 (60.7%)	0.3629
ASA score			
% 3 or 4	36/183 (19.7%)	25/135 (18.5%)	0.7962
CEA, ng/ml			
% with ≥ 200 ng/ml	16/178 (9.0%)	10/130 (7.7%)	0.6861
Primary tumour grade (poor vs well or moderate)			
% poor	10/176 (5.7%)	4/120 (3.33%)	0.3500
pTumour stage			
% 3 or 4	169/183 (92.4%)	124/135 (91.9%)	0.8705
pNode stage			
% 1 or 2	142/183 (77.6%)	86/135 (63.7%)	**0.0066**
Primary tumour (colon vs rectal)			
% colon	140/183 (76.5%)	109/135 (80.7%)	0.3648
Largest liver metastases			
% ≥ 5 cm diameter	37/182 (20.3%)	18/130 (13.9%)	0.1384
Distribution of liver metastases (bilobar vs unilobar)			
% bilobar	43/183 (23.5%)	42/133 (31.6%)	0.1097
Multiple vs solitary liver metastases			
% multiple metastases	76/183 (41.5%)	54/132 (40.9%)	0.9121
Synchronous vs metachronous			
% synchronous	89/183 (48.6%)	66/135 (48.9%)	0.964
Major vs minor			
% major	96/182 (52.7%)	59/133 (44.4%)	0.141
Margins (R1/R2 vs R0)			
% R0	151/183 (82.5%)	109/134 (81.3%)	0.789
DFI (> 12 months vs ≤ 12 months)			
% ≤ 12 months	132/183 (72.1%)	97/135 (71.9%)	0.956
Neoadjuvant chemotherapy for liver metastases			
% neoadjuvant chemotherapy	121/183 (66.1%)	71/134 (53%)	**0.018**
Adjuvant chemotherapy for liver metastases			
% adjuvant chemotherapy	69/179 (38.5%)	80/132 (60.6%)	**< 0.0001**
CRS liver score			
% with ≥ 3 points	75/183 (41.0%)	38/135 (28.2%)	**0.0181**

Overall, 228/318 (71.7%) patients had nodal metastases from primary colorectal cancer. 142/183 (77.6%) patients who had liver resection from P1 and 86/135 (63.7%) patients who had liver resection from P2 had primary colorectal cancer with nodal metastases. The difference between the proportion of patients with nodal metastases from both time periods was statistically significant (*p* = 0.0066).

113/318 (35.5%) of patients in the overall cohort had CRS score of ≥ 3 points. 75/183 (41.0%) patients who had liver resection from P1 and 38/135 (28.2%) patients who had liver resection from P2 had CRC score of ≥ 3 points. The difference between the proportion of patients with CRC score of ≥ 3 points from both time periods was statistically significant (*p* = 0.0181).

With regard to chemotherapy, 192/317 (60.6%) patients underwent neoadjuvant chemotherapy, and 149/311 (47.9%) patients underwent adjuvant chemotherapy. Between both time periods, a higher proportion of patients underwent neoadjuvant chemotherapy in P1, and this was statistically significant (*p* = 0.018). However, a higher proportion of patients had adjuvant chemotherapy from P2, and this was also statistically significant (*p* < 0.0001).

**Fig. 1:**
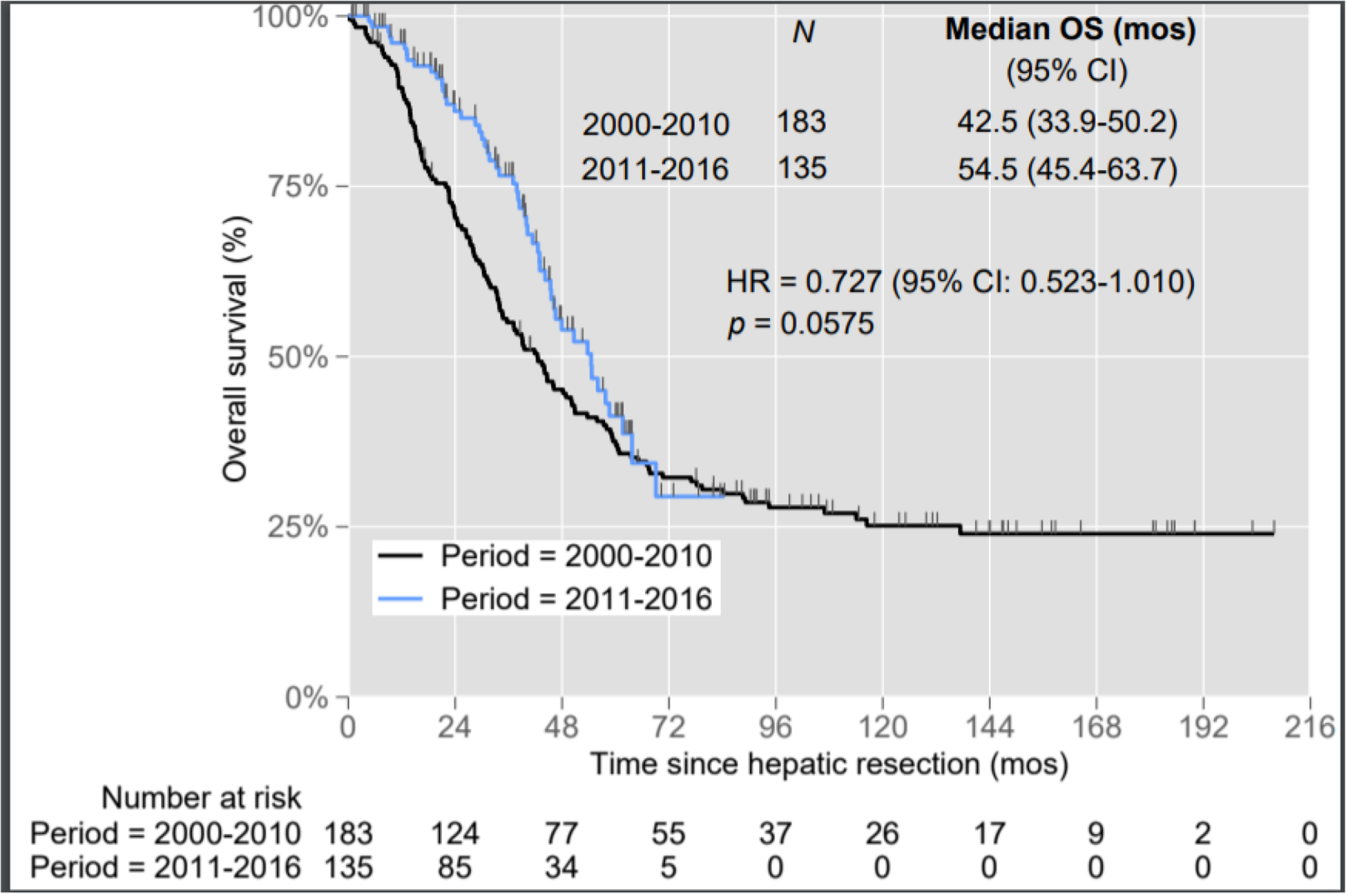
Kaplan Meir curve comparing 2011 to 2016 (P2) with 2000 to 2010 (P1). OS, overall survival; HR, hazard ratio; CI, confidence interval

The median overall survival for all patients was 46.5 months (95% CI 39.8–53.2 months). As shown in Fig. 1, the median overall survival was 54.5 months (95% CI 44.3–64.7 months) in P2 which was longer than P1 where median overall survival was 42.5 months (95% CI 33.5–51.4 months), and this difference approached statistical significance (*p* = 0.057). Table 2 summarizes the univariate survival analysis performed for the overall cohort. There was no difference in survival outcomes between both time periods with multivariate survival analysis (HR 0.850 95% CI 0.601–1.201, *p* = 0.357). Independent predictors of survival for the overall cohort included CEA levels ≥ 200 ng/ml, primary tumour grade and nodal metastases from primary colorectal tumour.

**Table 2:** Patient demographics and clinicopathological characteristics and their impact on OS in the overall cohort (*n* = 318)

Baseline variables	Unadjusted HR (95% CI)	***p*** value	Adjusted HR (95% CI)	***p*** value
Colorectal liver metastases diagnoses period				
2011 to 2016 vs 2000 to 2010	0.727 (0.523–1.010)	0.057	0.850 (0.601–1.201)	0.357
Age at resection, years				
(≥ 65 vs < 65 years)	0.756 (0.546–1.049)	0.094		
Sex				
Male vs female	1.192 (0.882–1.611)	0.252		
ASA score				
[3 and 4] vs [1 and 2]	1.172 (0.817–1.682)	0.389		
CEA, ng/ml				
% with ≥ 200 ng/ml	2.128 (1.359–3.332)	**0.001**	1.879 (1.182–2.986)	**0.008**
Primary tumour grade				
Poor vs well or moderate	2.365 (1.283–4.360)	**0.006**	3.017 (1.620–5.621)	**0.001**
pTumour stage				
3 and 4 vs 1 and 2	1.491 (0.847–2.624)	0.166		
pNode stage				
1 and 2 vs 0	2.021 (1.407–2.902)	**< 0.001**	2.098 (1.420–3.101)	**< 0.001**
Primary tumour				
Colon vs rectal	0.745 (0.517–1.075)	0.115		
Largest liver metastases				
≥ 5 vs < 5 cm	1.342 (0.935–1.927)	0.111		
Distribution of liver metastases				
Bilobar vs unilobar	1.196 (0.859–1.664)	0.289		
Multiple vs solitary liver metastases				
Multiple vs solitary	1.233 (0.917–1.658)	0.165		
Synchronous vs metachronous liver metastases				
Synchronous vs metachronous	1.463 (1.091–1.963)	**0.011**		
Major vs minor hepatectomy				
Major vs minor	1.473 (1.100–1.974)	**0.009**		
Margins (R1/R2 vs R0)				
R1/R2 vs R0	1.312 (0.899–1.915)	0.159		
DFI (> 12 months vs ≤ 12 months)				
> 12 months vs ≤ 12 months	0.708 (0.505–0.992)	**0.045**		
Neoadjuvant chemotherapy for liver metastases				
Yes vs no	1.455 (1.067–1.986)	**0.018**		
Adjuvant chemotherapy for liver metastases				
Yes vs no	0.834 (0.622–1.119)	0.226		
CRS liver score				
≥ 3 vs < 3	1.721 (1.281–2.311)	**< 0.0001**		

Table 3 summarizes the univariate survival analysis performed separately in the 2 time periods. Raised CEA levels ≥ 200 ng/ml, nodal metastases from primary colorectal cancer and CRS score ≥ 3 were associated with poor survival in both time periods, and there was no significant statistical difference upon comparison of their hazard ratios. Disease free interval (DFI) ≤ 12 months was associated with poorer overall survival in both time periods. In P1, DFI ≤ 12 months had a higher hazard ratio when compared to P2, and this difference was statistically significant (*p* = 0.0178). Poor primary tumour grade, synchronous liver metastases and neoadjuvant chemotherapy were associated with poorer overall survival in P1 but did not affect survival in P2. The differences in hazard ratios for these factors were not significant.

**Table 3:** Comparison of patient demographics and clinicopathological characteristics and their impact on OS between 2 time periods (2000 to 2010 vs 2011 to 2016)

Baseline variables	2000 to 2010 unadjusted HR (95% CI)	***p*** value	2011 to 2016 unadjusted HR (95% CI)	***p*** value	***p*** value*
Age at resection, years					
(≥ 65 vs < 65 years)	0.712 (0.482–1.051)	0.0873	0.920 (0.504–1.680)	0.7865	0.1084
Sex					
Male vs female	1.232 (0.866–1.752)	0.2462	1.173 (0.655–2.098)	0.5917	0.1381
ASA score					
[3 and 4] vs [1 and 2]	1.411 (0.937–2.124)	0.0990	0.631 (0.282–1.414)	0.2631	0.0521
CEA, ng/ml					
% with ≥ 200 ng/ml	1.796 (1.050–3.070)	**0.0324**	3.383 (1.511–7.573)	**0.0030**	0.1388
Primary tumour grade					
Poor vs well or moderate	2.417 (1.198–4.875)	**0.0137**	2.038 (0.639–6.504)	0.2291	0.1607
pTumour stage					
3 and 4 vs 1 and 2	1.929 (0.899–4.139)	0.0918	0.907 (0.387–2.125)	0.8214	0.0681
pNode stage					
1 and 2 vs 0	1.663 (1.065–2.595)	**0.0252**	2.923 (1.543–5.538)	**0.0010**	0.3195
Primary tumour					
Colon vs rectal	0.845 (0.571–1.250)	0.3998	0.899 (0.448–1.803)	0.7641	0.1671
Largest liver metastases					
≥ 5 vs < 5 cm	1.313 (0.867–1.988)	0.1979	1.306 (0.612–2.788)	0.4907	0.2049
Distribution of liver metastases					
Bilobar vs unilobar	1.142 (0.763–1.710)	0.5185	1.608 (0.888–2.914)	0.1171	0.1347
Multiple vs solitary liver metastases					
Multiple vs solitary	1.074 (0.757–1.524)	0.6898	1.959 (1.117–3.435)	**0.0190**	0.0950
Synchronous vs metachronous liver metastases					
Synchronous vs metachronous	1.428 (1.010–2.019)	**0.0441**	1.627 (0.932–2.842)	0.0870	0.1591
Major vs minor hepatectomy					
Major vs minor	1.400 (0.990–1.981)	0.0571	1.475 (0.853–2.552)	0.1647	0.2410
Margins (R1/R2 vs R0)					
R1/R2 vs R0	1.244 (0.798–1.940)	0.3344	1.473 (0.709–3.061)	0.2990	0.1776
DFI (> 12 months vs ≤ 12 months)					
> 12 months vs ≤ 12 months	0.300 (0.184–0.4904)	**< 0.0001**	0.472 (0.237–0.939)	**0.0327**	**0.0178**
Neoadjuvant chemotherapy for liver metastases					
Yes vs no	1.478 (1.005–2.173)	**0.0472**	1.340 (0.770–2.331)	0.3002	0.2496
Adjuvant chemotherapy for liver metastases					
Yes vs no	0.790 (0.553–1.128)	0.1945	1.126 (0.647–1.959)	0.6748	0.1604
CRS liver score					
≥ 3 vs < 3	1.478 (1.045–2.091)	**0.0273**	2.742 (1.538–4.888)	**0.0006**	0.2062

*p value for the interaction between study period and magnitude of hazard ratio was obtained by comparing regression coefficients from the Cox model

Table 4 summarizes the multivariate survival analysis performed separately in the 2 time periods. In P1, independent predictors of poor survival were poor tumour grade and nodal metastases. In P2, independent predictors of poor survival were multiple liver metastases and nodal metastases. These results demonstrate that nodal metastases from the primary colorectal cancer continue to remain an independent predictor of survival across the 2 time periods.

**Table 4:** Multivariable models of OS predictors after hepatectomy for CRC during 2000–2010 and 2011–2016

Predictors	Adjusted hazard ratio (95% CI)	***p*** value
**2000–2010 period**		
ASA ([3 and 4] vs [1 and 2])	1.455 (0.954–2.219)	0.082
Grade (poor vs well or moderate)	2.488 (1.191–5.199)	**0.015**
pNode (1 and 2 vs 0)	1.900 (1.201–3.006)	**0.006**
**2011–2016 period**		
pNode (1 and 2 vs 0)	2.626 (1.367–5.045)	**0.004**
Multiple vs solitary liver mets	1.878 (1.049–3.363)	**0.034**
CEA (≥ 200 ng/ml vs < 200 ng/ml)	2.223 (0.968–5.109)	0.060

With regard to the impact of neoadjuvant and adjuvant chemotherapy on survival outcomes, these variables were not included in the main multivariable models (Table 4) because the semi-automated stepwise elimination procedure did not identify them as independent predictors of survival. However, considering the possible impact of chemotherapy on survival outcomes, we made a clinically informed decision to conduct additional sensitivity analyses by forcing the inclusion of these variables in the multivariable models (Supplementary Table S1 and S2). In the overall cohort, adjuvant chemotherapy was associated with improved overall survival. Neoadjuvant chemotherapy was associated with poorer survival in P1 but had no impact on survival in P2. Adjuvant chemotherapy was associated with improved survival in P1 with no impact on survival in P2. The results of the sensitivity analyses (Supplementary Table S2) corroborated those of the main analyses (Table 4).

## Discussion

Resection of CLM has been established as the standard of care, and with modern chemotherapy, there is substantial improvement in overall survival which approaches 40 to 45% at 5 years [11]. Prior studies on resection of colorectal cancer liver metastases reviewed the effect of clinicopathological characteristics on survival outcomes [12, 13]. Miyoshi et al. showed that low rectal cancers, synchronous lung and liver metastases and T4a–4b disease were associated with poorer prognosis [14]. Mekenkamp et al. demonstrated that patients with synchronous CLM had poorer prognostic factors but no difference in median overall survival compared to patients with metachronous CLM [15]. Despite these studies on the effect of clinicopathological characteristics on survival, there are few studies that analyse the trends in the management of CLM over time which may provide valuable information on changes in clinical practice. A study from South Australia which compared outcomes from 1992 to 2005 and 2006 to 2015 showed better overall survival in the post 2006 era. This improvement in survival outcomes was attributed to better patient selection and improved perioperative management [16].

The development of the multidisciplinary tumour boards (MDTs) since 2011 was the basis of this study which aimed to evaluate the impact of the MDTs on patient selection and survival outcome. In this study, there were fewer patients with nodal metastases and high CRS scores who underwent curative surgery from P2 compared with P1. This could be attributed to multidisciplinary meetings which account for better patient selection. There was suggestion of improved survival in P2 compared with P1 as demonstrated in the univariate survival analysis. However, after adjusting for CEA levels, primary tumour grade and nodal metastases, there was no significant difference in survival between the 2 time periods. It is possible that the magnitude of the prognostic impact of variables used in the CRS differs, thus accounting at least partially for this.

Although liver resection for surgically operable CLM is associated with survival benefit, appropriate patient selection ensures that the appropriate treatment is given to each patient [17]. Technical resectability refers to the feasibility of obtaining a margin-negative resection while preserving adequate functional liver remnant (FLR) with vascular inflow and outflow and biliary drainage [18]. However, resectability of CLM should be determined with a multidisciplinary team with input from hepatobiliary surgeons, oncologists, radiologists and pathologists. In addition to technical considerations, there is increasing emphasis on oncological and prognostic evaluation prior to surgical resection. The aim is to select patients with better chance of cure or sustained disease remission with consideration of the underlying tumour biology. Traditional prognostic factors include characteristics of primary colorectal cancer such as the T stage and nodal status and characteristics of the CLM such as size, number of lesions, margin status as well as CEA levels and disease-free interval between primary colorectal cancer and development of the liver metastases [19, 20].

Clinically, the prognostic values of these individual factors are limited. The Fong clinical risk score (CRS) together with other scoring systems were developed to provide an overall risk assessment to predict long-term survival for patients prior to curative resection. However, these scores fail to demonstrate predictive accuracy for long-term survival, and their clinical utility remains uncertain [21, 22]. Similarly, in this study, CRS score was calculated, and a high score of ≥ 3 was associated with poor survival on univariate analysis demonstrated for the overall cohort and even across both time periods. However, with multivariate survival analysis, CRS score was not an independent predictor of survival.

Regardless, our study demonstrated that individual prognostic factors such as nodal metastases from primary colorectal cancer remain a strong independent predictive factor for overall survival in the overall cohort and even across both time periods. Interestingly, poorly differentiated colorectal cancer was an independent predictor for poor overall survival in P1, but it was not predictive for survival in P2. This could be due to smaller proportion of patients with poorly differentiated colorectal cancer in P2 compared to P1; however, the difference was not statistically significant.

Although there was similar proportion of patients with multiple liver metastases in both time periods, it was an independent predictor of overall survival in P2. This is supported by studies that demonstrate multiple liver metastases were associated with negative survival outcomes [5, 9]. This result also demonstrates that there are better survival outcomes for patients with solitary liver metastases in P2 compared with P1. This difference could be attributed to better surgical techniques, development of energy devices and increasing use of liver parenchyma sparing operations. Given that multiple liver metastases are a poor predictor of survival; this could be a contributing factor to no significant difference in survival outcomes being seen between the two time periods based on the multivariate survival analysis of the overall cohort. The implications to clinical practice would be that patients with multiple liver metastases which appear technically resectable would need to be assessed carefully for the oncological benefit of curative liver resection given that it is a poor predictor of overall survival.

Neoadjuvant and adjuvant chemotherapy was included in the multivariable model as shown in Supplementary Tables S1 and S2 to evaluate their prognostic impact on survival. Both neoadjuvant and adjuvant chemotherapy did not have a prognostic impact on survival in P2. However, neoadjuvant chemotherapy was a negative predictor of survival, and adjuvant chemotherapy was a positive predictor of survival in P1. Neoadjuvant chemotherapy is used to downstage unresectable CLM, and patients with good response to chemotherapy may subsequently undergo curative liver resection [23]. As patients selected for neoadjuvant chemotherapy may be associated with bulky metastatic disease, therefore this could explain its association with poor overall survival in P1. Adjuvant chemotherapy is used to reduce recurrence of CLM after liver resection by eradicating residual micro-metastases. However, a systematic review showed no significant improvement in median overall survival in patients who underwent chemotherapy and surgery compared with surgery alone [24]. In another study, adjuvant chemotherapy post resection of CLM had a 54% recurrence free survival and 55% overall survival advantage compared to surgery alone [25]. Although patients in P1 who had adjuvant chemotherapy had improved overall survival, the same prognostic impact was not demonstrated in P2. This was in spite of a higher proportion of patients undergoing adjuvant chemotherapy in P2. A possible explanation would be that better patient selection and advances in surgical techniques in P2 contributed to the reduced prognostic impact of adjuvant chemotherapy on survival in this time period.

This study has evaluated the trends in management of CLM as well as the changes in prognostic factors over the 2 time periods. This has provided information on clinical practice that may influence patient selection for curative resection and treatment strategies. As patients with multiple liver metastases or known nodal metastases were associated with poorer survival, they will likely have more frequent surveillance scans and advised to undergo adjuvant chemotherapy to improve survival. However, the limitations of this study would include the small sample size which upon subgroup survival analysis result in the study being under powered, and therefore, the predictive value of other prognostic factors may not have been identified. Another limitation would be the presence of missing data which will affect the sample size for survival analysis. Given the retrospective observational nature of this study from a single centre, the results are not entirely representative. Additionally, the details of both neoadjuvant and adjuvant chemotherapy regimens were not collected and may not be standardized, and this could confound the results of the survival analysis. However, this study highlights the need for further multicentre trials or population-based cohort studies to properly evaluate the changes in trends and management of CLM over time.

## Conclusion

In conclusion, nodal metastases from primary colorectal cancer continue to remain an independent predictor of poor survival across time for patients who undergo curative liver resection for colorectal liver metastases. Although there are changes in patient selection over time, there is no difference in survival outcomes between the two time periods. Therefore, patients with multiple liver metastases should be carefully evaluated prior to curative liver resection given that it is also an independent predictor of overall survival.

## Supplementary information

**Additional file 1: Supplementary Table S1.** Multivariable model of patient demographics and clinicopathological characteristics and their impact on OS in the overall cohort (n = 318) with inclusion of neoadjuvant and adjuvant chemotherapy. **Supplementary Table S2.** Multivariable models of OS predictors after hepatectomy for CRC during 2000-2010 and 2011-2016 with inclusion of neoadjuvant and adjuvant chemotherapy

## Data Availability

Not applicable
