# Supplementary material for: Impact of multidisciplinary tumour boards (MTB) on the clinicopathological characteristics and outcomes of resected colorectal liver metastases across time
Source: World J Surg Oncol. 2020 Sep 3;18:237. doi: 10.1186/s12957-020-01984-8 (PMC7650267; doi:10.1186/s12957-020-01984-8)
Supplement: Supplementary file 1 — Additional file 1: Supplementary Table S1. Multivariable model of patient demographics and clinicopathological characteristics and their impact on OS in the overall cohort (n = 318) with inclusion of neoadjuvant and adjuvant chemotherapy. Supplementary Table S2. Multivariable models of OS predictors after hepatectomy for CRC during 2000-2010 and 2011-2016 with inclusion of neoadjuvant and adjuvant chemotherapy [file 12957_2020_1984_MOESM1_ESM.docx]

| **Supplementary Table S1. Multivariable model of patient demographics and clinicopathological characteristics and their impact on OS in the overall cohort (n = 318) with inclusion of neoadjuvant and adjuvant chemotherapy** | | | | | |
| --- | --- | --- | --- | --- | --- |
| **Baseline variables** | **Unadjusted HR (95% CI)** | ***P* value** | | **Adjusted HR**  **(95% CI)** | ***P* value** |
| Colorectal liver metastases diagnoses period | |  | |  |  |
| - 2011 to 2016 vs 2000 to 2010 | 0.727 (0.523 – 1.010) | 0.057 | | 0.915 (0.643 -1.301) | 0.620 |
| CEA, ng/ml |  |  | |  |  |
| - % with ≥ 200 ng/ml | 2.128 (1.359-3.332) | **0.001** | | 1.765(1.103 – 2.826) | **0.018** |
| Primary tumour grade |  |  | |  |  |
| - Poor vs well or moderate | 2.365 (1.283-4.360) | **0.006** | | 3.103 (1.663 – 5.789) | **<0.001** |
| pNode stage |  |  | |  |  |
| - 1 & 2 vs 0 | 2.021 (1.407-2.902) | **<0.001** | | 2.129 (1.426 – 3.180) | **<0.001** |
| Neoadjuvant chemotherapy for liver metastases | | |  |  |  |
| - Yes vs no | 1.455 (1.067-1.986) | **0.018** | | 1.344 (0.949 – 1.905) | 0.096 |
| Adjuvant chemotherapy for liver metastases | |  | |  |  |
| - Yes vs no | 0.834 (0.622-1.119) | 0.226 | | 0.743 (0.536 – 1.001) | 0.051 |

| **Supplementary Table S2. Multivariable models of OS predictors after hepatectomy for CRC during 2000-2010 and 2011-2016 with inclusion of neoadjuvant and adjuvant chemotherapy** | | |
| --- | --- | --- |
| **Predictors** | **Adjusted Hazard ratio†** | ***P* Value** |
| **2000-2010 period** | |  |
| ASA ([3 and 4] vs [1 and 2]) | 1.545 (1.003 – 2.381) | **0.048** |
| Grade (poor vs well or moderate) | 2.454 (1.168 – 5.155) | **0.018** |
| pNode (1 & 2 vs 0) | 1.859 (1.154 – 2.995) | **0.011** |
| Neoadjuvant chemotherapy | 1.664 (1.082 – 2.560) | **0.021** |
| Adjuvant chemotherapy | 0.629 (0.429 – 0.924) | **0.018** |
| **2011-2016 period** | |  |
| pNode (1 & 2 vs 0) | 3.579 (1.330 – 5.002) | **0.005** |
| Multiple vs solitary liver mets | 1.798 (0.965 – 3.350) | 0.065 |
| CEA (≥ 200ng/ml vs <200ng/ml) | 2.440 (1.026 – 5.805) | **0.044** |
| Neoadjuvant chemotherapy | 1.012 (0.553 – 1.852) | 0.969 |
| Adjuvant chemotherapy | 1.221 (0.672 – 2.220) | 0.513 |
|  | | |
